# DAIS-MQTT: A Distributed MQTT Communication Method Based on Intelligent QoS Routing and Hierarchical Collaboration

**DOI:** 10.3390/s26113564

**Published:** 2026-06-03

**Authors:** Mengjia Lian, Wanda Yin, Anying Chai, Ping Huang, Yunpeng Sun, Enqiu He

**Affiliations:** 1 School of Mathematics and Information Engineering, Longyan University, Longyan 364012, China; 2 College of Information Science and Engineering, Shenyang University of Technology, Shenyang 110870, China; 3 School of Electrical Engineering, Shenyang Institute of Engineering, Shenyang 110136, China; 4 School of Chemical Equipment, Shenyang University of Technology, Liaoyang 111000, China

**Keywords:** Industrial Internet of Things, data scheduling, MQTT, distributed, adaptive routing

## Abstract

The continuous growth of IIoT systems has significantly increased the number of connected devices and message interactions, creating higher requirements for communication mechanisms in terms of scalability and adaptability under dynamic network environments. Although MQTT is widely used for its lightweight communication, its traditional centralized broker architecture limits scalability and fault tolerance in large-scale data transmission, reducing system scalability and fault tolerance. Additionally, static QoS configuration is difficult to adapt to dynamic environmental changes, resulting in high end-to-end latency and limited system throughput. To address these issues, this paper proposes a distributed MQTT communication method based on intelligent QoS routing and hierarchical collaboration (DAIS-MQTT). This method designs a network routing algorithm based on a hierarchical tree structure (LCN), which effectively addresses the scalability limitation of centralized proxies by enabling multi-level proxy collaboration and self-recovery from faults. At the same time, it proposes a QoS routing algorithm based on intelligent decision trees (IQR), which jointly optimizes proxy selection and QoS levels to dynamically adapt to changes in the network environment, thereby solving the problem of insufficient adaptability in static QoS configurations. Experimental results show that compared with the traditional MQTT-based communication method, the DAIS-MQTT method reduces the average message delay by 29.9%, increases system throughput by 28.2%, and maintains a reliable transmission rate of 98.7% in unreliable network environments, making it suitable for high-dynamic and large-scale IIoT communication scenarios.

## 1. Introduction

The ongoing technological revolution and industrial transformation are driving the deep integration of emerging information and communication technologies—such as the Internet of Things (IoT), cloud computing, and artificial intelligence—propelling the manufacturing industry toward unprecedented levels of automation and intelligence [1]. As a key supporting technology for intelligent manufacturing, the Industrial Internet of Things (IIoT) connects a large number of heterogeneous devices, enabling real-time perception, intelligent collaboration, and remote control of industrial production processes. It has become an important engine for promoting industrial digital transformation [2,3]. Through the collaborative integration of the perception layer, network layer, and application layer, IIoT not only realizes dynamic resource scheduling and optimization but also supports equipment status prediction and flexible production control, significantly enhancing the agility and intelligence of industrial systems. With the explosive growth of device connection scales and the continuous increase in business logic complexity, industrial communication systems are facing more severe performance challenges: they must support the high concurrent access of massive devices while ensuring the low latency, high throughput, and high reliability of real-time data transmission [4]. Current communication modes perform poorly in complex network environments. They are also not suitable for fluctuating network loads.

The lightweight messaging protocol MQTT is widely used in the Industrial Internet of Things (IIoT). Many researchers adopt MQTT because it uses a publish/subscribe model, is easy to implement, and consumes few resources [5]. Most traditional MQTT systems are based on a centralised Broker. A single broker node in this architecture will be used to gather and distribute all messages. With the rapid increase in the number of connected devices, the centralised architecture is likely to be slow. Therefore, the system is not feasible. It also increases the risk of a single point of failure [6,7]. Network conditions in actual factories are often unstable. Fluctuations in links and node congestion and other conditions of high-communication demand can all lead to network instability. MQTT will not be in a reliable real-time messaging mode under the above circumstances. MQTT is used to realise either static polling or random forwarding for multi-topic communication. These methods are unable to modify the route decision in response to changes in the network or topic load. Therefore, some high-demand topics may be overloaded and others will remain unused by the network. This disparity results in poor resource utilisation and lower-efficiency communication overall [8,9]. The QoS mechanism in MQTT also has limitations. Most MQTT systems use predefined QoS settings. These settings usually cannot adapt dynamically to changes in link conditions. Therefore, the system often struggles to balance transmission reliability and communication efficiency. This issue further affects the timeliness and robustness of message communication.

In recent years, many studies have been conducted on distributed message transmission mechanisms for complex network environments to address the above problems. The Load of communication will be spread over multiple brokers. Edge computing can be added to MQTT systems. The above method can move message processing closer to the data source to improve system availability and response speed [10].

Some optimisation work has been carried out on the MQTT-based communication model in previous studies. However, there are still many problems in large-scale IIoT environments. First, many broker selection methods are still based on preset rules or limited local state information. The above methods may not be suitable for dealing with changes in network delay and resource conditions. Thus, the Communication Load will be distributed unevenly. This problem will also reduce routing and thus lead to a performance drop [11]. The second is that most topic mapping methods fail to consider the combined effects of historical traffic patterns, message features, and real-time network conditions fully. It will thus reduce the economy of resources. Some popular subjects are too popular and crowded, while other resources are not used. Third, most of the existing QoS strategies do not consider changes in network link conditions in real time. Therefore, these strategies cannot adjust the service level flexibly according to the current communication environment. Therefore, the actual service quality will not meet the transmission requirements. This problem will also reduce the system’s throughput and communication reliability [12,13,14]. Therefore, a high-availability communication framework is needed for IIoT systems. Several network-awareness features, an elastic broker-topic selection function, and adjustable QoS control need to be added to the framework. The above attributes will help ensure the stable operation of a large-scale network.

This paper introduces a distributed MQTT communication mode called DAIS-MQTT. The method is intelligent QoS routing and hierarchical cooperation. DAIS-MQTT is a distributed multi-agent system. The two components of the intelligent QoS routing algorithm (IQR) and the layered collaborative network routing algorithm (LCN) have been integrated. The proposed approach will be more adaptable to network changes and achieve better communication performance in complex networks. DAIS-MQTT is distributed and can coordinate resources through a communication manager. The above design can help the system achieve more effective topology awareness and load balancing in large-scale IoT environments.

Unlike existing distributed MQTT systems that mainly focus on either topology-aware broker deployment or static QoS adaptation, DAIS-MQTT introduces a unified framework that jointly integrates hierarchical broker collaboration and adaptive QoS-aware routing. At the architectural level, the proposed framework combines decentralized state synchronization, hierarchical topology management, and intelligent routing decision-making into a coordinated three-layer communication system. At the algorithmic level, the proposed IQR mechanism jointly selects brokers. It optimizes QoS through adaptive decision-tree-based routing, while the LCN mechanism provides topology-aware hierarchical routing and decentralized fault recovery. In contrast to existing approaches that mainly introduce incremental improvements to broker deployment or static routing policies, DAIS-MQTT emphasizes the collaborative integration of adaptive routing, hierarchical coordination, and dynamic QoS regulation within a unified distributed MQTT framework.

The principal contributions of this paper are listed as follows:

(1) We propose a distributed adaptive communication framework called DAIS-MQTT for large-scale Industrial Internet of Things environments. The framework includes a network infrastructure layer, an agent collaboration layer, and an intelligent decision-making layer. The proposed framework jointly integrates hierarchical broker collaboration, decentralized state synchronization, and adaptive QoS-aware routing within a unified architecture, enabling coordinated optimization beyond conventional approaches that treat broker deployment and QoS adaptation separately. The proposed framework also reduces the scalability, reliability, and adaptability limitations of traditional MQTT architectures.
(2) We propose an intelligent QoS routing algorithm called IQR based on a decision-tree structure. The algorithm jointly optimizes broker selection and QoS configuration in MQTT communication. We design the algorithm to dynamically adjust routing strategies and QoS levels based on historical communication performance and current network conditions. We also integrate message topics, broker nodes, and QoS parameters into a hierarchical decision process. This design improves resource utilization and enhances overall system performance.
(3) We further design a hierarchical tree-based routing algorithm called LCN to support distributed collaborative communication. The algorithm organizes broker nodes through RTT-based hierarchical classification and decentralized state propagation. We also maintain subscription information through topic routing tables. In addition, we integrate a health monitoring mechanism into the routing process. This design enables fast fault detection and recovery. The proposed approach, therefore, improves system robustness and adaptability in dynamic network environments.

The rest of this paper is organized as follows. Section 2 reviews related research on communication optimization in the Industrial Internet of Things. This section discusses the limitations of traditional MQTT architectures, distributed message transmission methods, and intelligent scheduling and adaptive control techniques used in communication systems. Section 3 introduces the architecture of the proposed DAIS-MQTT framework. This section also explains the functions and interactions of each layer and provides detailed descriptions of the implementation of the IQR and LCN algorithms. Section 4 evaluates the performance of DAIS-MQTT through experiments, focusing on latency, throughput, and reliability under different network conditions, and compares the proposed method with existing MQTT-based frameworks. Finally, Section 5 summarizes the main contributions of this work and outlines possible future research directions.

## 2. Related Work

With the rapid development of IIoT, the types of devices in industrial scenarios are increasingly diverse, and the scale of connections continues to expand. While communication systems need to accommodate the access of a large number of heterogeneous devices, they also face multiple challenges in terms of real-time performance, reliability, and flexibility [15,16]. Azari et al. [17] established an interference model and derived expressions for reliability and energy consumption for multi-technology coexisting IoT environments, analyzing the impact of communication parameters on device lifespan and performance. Compared to traditional connection-oriented communication protocols, lightweight message protocols based on the publish/subscribe mechanism demonstrate greater adaptability and deployment efficiency in IIoT scenarios. Ferraz Junior et al. [18] proposed a message structure optimization method for the Internet of Things, standardizing the publish-subscribe topics and payloads to achieve context awareness and end-to-end security. Experiments have shown that this structure is applicable to AMQP, DDS, and MQTT protocols, reducing transmission bytes and improving communication efficiency for ultra-low-power and high-capacity devices, while ensuring confidentiality, integrity, and authenticity between devices and cloud platforms. Among these protocols, MQTT has become one of the main data transmission protocols in industrial environments. The protocol requires low bandwidth overhead, supports simple implementation, and provides good cross-platform compatibility [19]. MQTT simplifies the interaction logic between terminal nodes by introducing an intermediate broker for message decoupling and forwarding, making it suitable for high-frequency data interaction in resource-constrained device environments. It is widely used in critical tasks such as industrial monitoring, remote control, and status synchronization [20].

However, with the continuous surge in the number of access terminals and the significant increase in message interaction frequency in industrial applications, the traditional MQTT communication model is facing obvious performance bottlenecks. On the one hand, the centralized proxy architecture is prone to single-point failures and communication congestion, making it challenging to meet the system’s stability and scalability requirements in high-concurrency scenarios. On the other hand, the MQTT protocol itself has limited support for topic management, transmission paths, and quality of service control, and lacks mechanisms for perception and adaptation to dynamic changes in complex industrial network environments. Therefore, optimizing the MQTT communication architecture based on existing applications to enhance its concurrent performance and stability in industrial scenarios has become one of the current key research directions [21]. Chai et al. [22] addressed the concurrency and throughput limitations of traditional IIoT communication systems by proposing a distributed high-availability MQTT model (DUA-MQTT) based on the OPC UA architecture. By integrating distributed MQTT, they enhanced data transmission concurrency. Additionally, they designed an information-modeling model (MAC-GC) that generates OPC UA-compliant structured data nodes through hierarchical annotation to accurately map device functions and attributes.

To alleviate the performance bottlenecks faced by traditional centralized MQTT architectures in industrial IoT, researchers have proposed various distributed architecture solutions to enhance the system’s scalability and concurrent processing capabilities [23]. Liu et al. [24] proposed a distributed resource sharing mechanism based on ADMM, which introduced SDN to support efficient collaboration of edge resources in IIoT, ensuring the benefits of both devices and edge servers while improving system stability and convergence efficiency. Longo et al. [25] developed the BORDER benchmarking framework to evaluate MQTT performance in distributed broker environments. The framework uses simulators and Docker-based deployments for testing. Their work provides useful support for later studies on protocol optimization. Hmissi et al. [26] designed a transparent distributed MQTT proxy connection mechanism. The mechanism allows subscribers to communicate without knowing the broker’s information in advance. This design reduces communication latency and improves transmission efficiency in distributed systems. Park et al. [27] proposed an enhanced MQTT protocol based on an SDN multicast mechanism. Their method removes the strong dependence on a centralized Broker. The system builds a bidirectional multicast tree for message forwarding. This approach reduces transmission delay and network overhead. It also improves communication efficiency in large-scale IoT environments. Buccafurri et al. [28] proposed the MQTT-A protocol. The protocol integrates a peer-to-peer collaboration mechanism to improve anonymity and security during data transmission. This design is suitable for industrial applications with strict privacy requirements. In addition to communication performance optimization, routing security and privacy protection have become important research topics in IIoT communication systems. Zhu et al. [29] proposed the FLAG-POR protocol for MONs. Their method uses a group-based pairing-onion routing approach. The protocol improves forwarding efficiency through collaborative relay mechanisms. The method also provides forward security during communication. Although their work mainly focuses on anonymity protection, the group-based forwarding strategy is similar to the hierarchical broker collaboration mechanism used in this study. However, our work mainly focuses on adaptive QoS optimization and communication efficiency. These security-oriented studies complement existing routing optimization methods by improving data protection and user privacy during message transmission. Kurte et al. [30] further proposed an extended DSF-IoT mechanism that introduces a delegation-based interaction method between constrained and unconstrained devices, enabling the construction of a decentralized heterogeneous IoT environment. They also implemented a gateway prototype for evaluation, and the experimental results showed that the mechanism can operate reliably on resource-constrained devices while maintaining trust and privacy guarantees. Bartoli et al. [31] proposed an SDN architecture for industrial IoT, combining the MQTT-SN protocol to adapt to resource-constrained devices, optimizing end-to-end latency, and enhancing communication efficiency. This approach supported an extended publish/subscribe mechanism and was verified in practical scenarios for its low latency and low overhead characteristics, making it suitable for data flow management in heterogeneous industrial applications. Wu et al. [32] proposed a dual-blockchain distributed data management framework, MapChain-D, for large-scale IIoT, which introduced a mapping mechanism between data chains and index chains to achieve efficient storage and retrieval. Experiments in LoRaWAN and Ethereum environments demonstrated that this method had good scalability and resource adaptability, making it suitable for scenarios with resource-constrained edge devices.

Although the above-mentioned methods have alleviated the problem of single-node overload to some extent, due to the lack of real-time perception of network status and communication load, it remains difficult to achieve a dynamic and balanced distribution of proxy resources. Especially in industrial field environments where communication density and data traffic fluctuate frequently, static proxy mapping strategies often result in some nodes being overloaded while others remain idle, leading to a waste of system resources and increased communication latency. At the same time, existing distributed architectures are mostly based on the independent operation of nodes and lack cross-proxy collaboration mechanisms. Message routing paths are difficult to optimize dynamically based on the system’s global state, which limits overall improvements in communication efficiency. Therefore, constructing a distributed broker collaboration mechanism with real-time feedback capability and adaptive path selection strategies has become an important research direction for improving the performance and stability of MQTT systems.

In MQTT-based industrial communication systems, topics serve as the fundamental element of the publish/subscribe model, and their management directly influences communication efficiency and system load-balancing performance. De Rango et al. [33] proposed the DLST-MQTT security mechanism, which introduces a dynamically switchable topic security layer to support end-to-end secure communication. Their approach reduces the communication burden on the Broker while improving both system flexibility and security. Zambrano et al. [34] developed the SiGPro system using topic management and Bridge mechanisms. The system supports a progressive notification and topic-distance-aware mode. This Design will improve communication. Improve the real-time performance of the system’s missing-person location in a multi-city environment.

Recently, out of the need to improve system adaptability, various dynamic topic-redistribution methods have been proposed. The above ways usually consider the number of historical messages, publication speed, and queue size. Some ways have also added message priority and service attributes to the weighted control. Although these methods improve communication performance to some extent, two major problems remain. First, most existing methods mainly focus on optimization at the individual topic level. These methods lack global collaborative modeling across the entire system. Second, most methods lack real-time feedback mechanisms to adapt to changing network conditions. As a result, the system may struggle to maintain stable transmission performance and high communication efficiency under heavy network load. Therefore, communication systems still require a topic distribution mechanism with multidimensional performance awareness and adaptive feedback regulation. Such a mechanism can support finer-grained resource management and improve overall communication efficiency collaboratively.

QoS is an important feature of the MQTT protocol. The protocol provides three predefined service levels, including QoS 0, QoS 1, and QoS 2. These service levels support different requirements for transmission reliability and communication latency in various applications. Traditional MQTT systems usually determine QoS configurations through static rules. However, Industrial Internet of Things environments often contain unstable links, network congestion, and heterogeneous devices. Therefore, the static QoS setting is not suitable for the new environment. Mukherjee et al. [35] have put forward a hierarchical message transmission framework for Social IoT environments based on flying ad hoc networks. Their framework combines opportunistic routing with 6G SDN slicing technology. The Design has improved the efficiency of communication and resource allocation under QoS Level 2 service. It will include full management functions for large-scale events and emergencies. Guha Roy et al. [36] have proposed a secure communication platform based on blockchain technology and hash-based encryption. Their method uses an MQTT Broker for cloud data transmission. The framework also supports QoS monitoring and resource recovery through a mobile application framework. This design improves communication security and system reliability. Motamedi et al. [37] addressed the issue of MQTT communication efficiency in the smart greenhouse scenario by proposing a message classification architecture. By standardizing topic classification and payload formats, they improved the processing performance and scalability of the Broker under high-concurrency subscription conditions from multiple clients, optimizing message transmission efficiency and system response speed. In addition to QoS optimization, recent studies have also explored the combination of QoS management and privacy-aware routing strategies. Wang et al. [38] proposed the QoSPR routing protocol for 5G-enabled IIoT environments. The protocol considers both QoS requirements and privacy protection. Their method applies federated reinforcement learning to optimize gateway deployment. This design helps reduce communication latency and improve load-balancing performance. However, QoSPR mainly focuses on gateway placement optimization. The protocol does not emphasize the joint adjustment of broker selection and QoS levels at the topic and message level. In contrast, our method focuses on fine-grained collaborative optimization of broker selection and QoS configuration under dynamic network conditions. Our work also places greater emphasis on communication efficiency than on infrastructure deployment or privacy-oriented routing mechanisms.

To further enhance service adaptability, recent research has gradually focused on QoS adaptive adjustment mechanisms based on real-time network performance indicators, dynamically adjusting message service levels to balance transmission efficiency and critical data protection [39]. Although such methods have significantly improved system stability and resource utilization in dynamic environments, they still generally have the following problems: reliance on a single indicator, lack of system-wide collaborative optimization, and additional overhead caused by frequent QoS adjustments. Therefore, constructing a QoS adaptive mechanism that integrates multi-source performance feedback and multi-objective optimization has become an important research direction in industrial MQTT communication.

Most existing studies treat routing optimization and QoS adaptation as separate problems. Only a small number of methods consider jointly optimizing routing decisions and QoS selection in dynamic network environments. In addition, many current solutions still rely on centralized architectures or loosely coordinated distributed mechanisms. These limitations reduce system scalability and adaptability in large-scale IIoT environments.

In response to the centralized proxy limitations on system scalability and fault tolerance, as well as the static QoS configuration’s difficulty in adapting to dynamic environments in the traditional MQTT communication model in large-scale distributed Internet of Things (IoT) environments, this paper proposes a distributed MQTT message communication method, DAIS-MQTT, based on intelligent QoS routing and hierarchical collaboration. This method is based on a three-layer functional architecture, integrating the intelligent QoS routing algorithm (IQR) and the hierarchical collaboration network routing algorithm (LCN), which achieves an intelligent proxy selection strategy and a dynamic QoS level optimization mechanism, thereby effectively enhancing the system’s scalability and network adaptability. Meanwhile, this paper proposes a hierarchical tree-like scheduling mechanism, which constructs an efficient distributed collaborative architecture through decentralized state synchronization and RTT-based hierarchical division. Through the collaborative optimization of intelligent routing decisions and adaptive service quality, DAIS-MQTT significantly reduces message transmission delay in complex, heterogeneous network environments, improves system throughput and reliability, and meets the requirements of large-scale IoT applications for efficient and stable message transmission.

Existing distributed MQTT systems, such as DUA-MQTT, primarily improve scalability through distributed broker deployment and protocol integration, whereas topology-aware approaches, such as TOD-MQTT, primarily focus on topology optimization and routing efficiency. The proposed DAIS-MQTT framework differs from these approaches in several aspects. First, the framework introduces a unified hierarchical collaboration architecture with decentralized state synchronization, enabling coordinated routing and topology management across distributed broker nodes. Second, the IQR mechanism jointly optimizes broker selection and QoS configuration via adaptive decision-tree-based routing, thereby coordinating routing decisions and QoS management within a unified optimization process. Third, the LCN mechanism combines topology-aware hierarchical scheduling with dynamic fault recovery and adaptive state propagation, enabling collaborative optimization under dynamic network conditions. Therefore, the proposed method can be regarded as an integrated distributed MQTT communication framework that combines architectural coordination and adaptive decision-making rather than solely introducing incremental routing optimization.

## 3. A Distributed MQTT Communication Method Based on Intelligent QoS Routing and Hierarchical Collaboration

In this section, a distributed MQTT communication method based on intelligent QoS routing and hierarchical collaboration, namely Distributed Adaptive Intelligent Scheduling for MQTT (DAIS-MQTT), is systematically introduced. This method, proposed in this paper, is based on intelligent QoS routing and hierarchical collaboration. DAIS-MQTT achieves efficient communication through a three-layer architecture. The network infrastructure layer is responsible for discovering topology and monitoring status, ensuring the stability of the infrastructure. The agent collaboration layer realizes efficient message transmission through decentralized status synchronization and load balancing. The intelligent decision-making layer combines the IQR and LCN algorithms to optimize path selection and QoS configuration, ensuring low-latency transmission of critical data. Through the collaborative work of these three layers, DAIS-MQTT enhances the system’s communication efficiency and adaptability in dynamic environments.

### 3.1. DAIS-MQTT Overall Architecture

**Figure 1:**
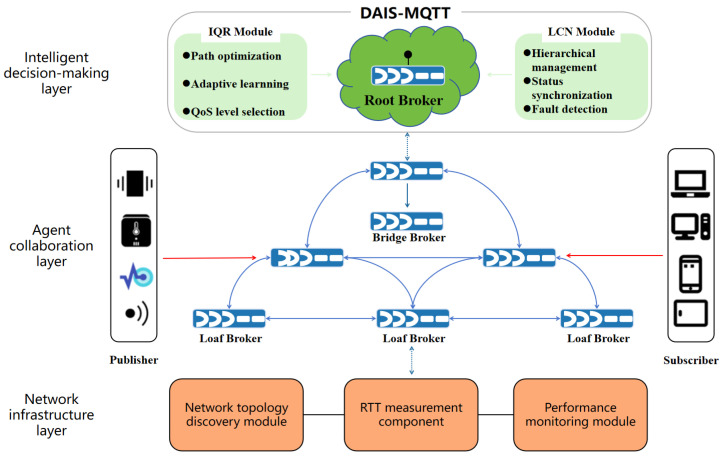
DAIS-MQTT Network Architecture Diagram.

DAIS-MQTT incorporates a distributed architecture, intelligent decision-making, and adaptive control mechanisms to establish a message transmission method with high scalability and robust autonomous adaptability. In its overall architecture, DAIS-MQTT combines three major mechanisms, namely intelligent routing decision-making, decentralized collaborative control, and adaptive QoS regulation, to improve message transmission efficiency in complex and dynamic IoT environments. As shown in Figure 1, the framework comprises three cooperative functional layers: the network infrastructure layer, the agent collaboration layer, and the intelligent decision-making layer. The network infrastructure layer manages the underlying network topology and connection conditions in the deployment environment. This layer provides the basic support for system operation. The multiple distributed MQTT agent nodes are in the agent collaboration layer. Together, these nodes will conduct message caching, relaying, and forwarding. The intelligent decision-making layer acts as the central scheduling component of the framework. This layer dynamically selects transmission paths and adjusts QoS levels based on network conditions, load distribution, and message characteristics. The framework can still use MQTT and is, therefore, convenient to deploy flexibly; it is also protocol-transparent.

The network infrastructure layer is responsible for topology discovery, RTT measurement, and network performance monitoring. Continuously collect topological information of currently active agent nodes and their connections in the topology discovery module. This module will update the system’s display of the network structure. The RTT measurement module periodically measures the round-trip delay at each node. The module will then provide latency information for building a network distance matrix. At the same time, a performance monitoring module will also record the above network indicators, such as link delay, packet loss rate, and bandwidth utilisation. The module will inform the agent collaboration layer of any network-state changes by emitting an event. Together, the above modules are used by the system to observe network conditions in real time. The system can also respond quickly to network fluctuations.

The agent collaboration layer is the intermediary hub of DAIS-MQTT. There are several distributed MQTT agent nodes in this layer. A layer that uses a decentralized state-synchronization mode. Therefore, the agent nodes can communicate information such as subscribed topics, load status, and health among themselves. This design enables self-organizing management of the cluster. In the message transmission process, the agent collaboration layer is responsible for receiving messages from publishers, selecting the appropriate forwarding path based on the routing instructions from the intelligent decision-making layer, and delivering the messages to the target subscribers. Additionally, this layer implements load balancing and failover functions among agents, ensuring the overall stability and reliability of the system by dynamically adjusting the message distribution strategy.

The intelligent decision-making layer is the core control center of DAIS-MQTT, integrating the QoS routing algorithm based on intelligent decision trees (IQR) and the hierarchical cooperative network routing algorithm (LCN), and combining the message priority management mechanism to achieve global optimization control. This layer constructs a joint decision-making model and performs real-time optimization calculations based on state data, such as link delay and packet loss rate, collected by the network infrastructure layer, as well as load and health indicators provided by the agent collaboration layer. During message processing, the IQR algorithm formalizes the MQTT routing decision as a joint optimization of agent selection and QoS level, and dynamically allocates the optimal transmission path and QoS level for different message types through adaptive learning. The LCN algorithm achieves topology-aware distributed routing collaboration and rapid fault recovery based on the hierarchical division of RTT and decentralized state propagation. Ultimately, the intelligent decision-making layer issues the optimization results to the agent collaboration layer for execution, and continuously adjusts the decision parameters by maintaining a historical performance database and a closed-loop feedback mechanism, thereby enhancing the adaptability, robustness, and global performance of the system in a dynamic network environment. This interaction establishes a dynamic feedback loop between decision-making and execution, which is fundamental to the overall system coordination.

Through the cooperation of these three layers, DAIS-MQTT supports a complete processing flow from network perception and agent coordination to intelligent decision-making. The design can provide a high-efficiency, high-reliability, and highly adaptable communication solution for MQTT message transmission in the Internet of Things (IoT). The system is also compatible with the standard MQTT protocol. At the same time, it will improve communication effectiveness and service quality assurance in a complex network.

**Figure 2:**
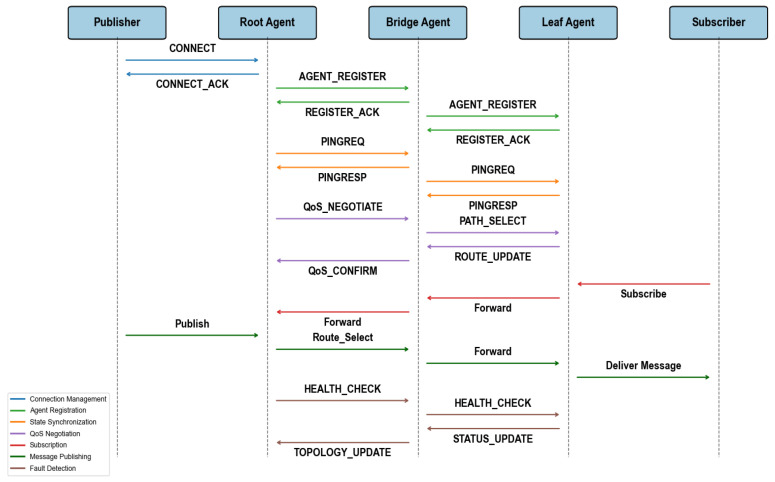
DAIS-MQTT Communication Flowchart.

Figure 2 shows the full communication workflow of DAIS-MQTT. The Publisher initiates the connection to the Root Agent and begins the process. The system will perform the initial handshaking using CONNECT and CONNECT_ACK messages at this time. After a successful connection, the system will open the agent registration module. At this time, the Root Agent, Bridge Agent, and Leaf Agent build a distributed network topology via AGENT_REGISTER and REGISTER_ACK messages. A three-level agent architecture is thus formed.

The Network Topology has been set up, and now we will begin state synchronisation. The above is a basic function of DAIS-MQTT. Agent nodes send status information via PINGREQ and receive PINGRESP. Exchanged Information: node load, network latency, health status, etc. The above data support the real-time intelligent routing decision. Next, the system will be in the IQR algorithm stage. The Root Agent and Bridge Agent perform QoS_NEGOTIATE operations at this time. The Bridge Agent and Leaf Agent then run PATH_SELECT to select the routing path. Send ROUTE_UPDATE messages in the system to update routing information. Finally, the system sets the QoS level in QoS_CONFIRM. The above steps will realise the main purpose of the IQR intelligent QoS routing algorithm.

When a Subscriber sends a Subscribe request, the message is forwarded upward through the agent hierarchy. The message passes through the Leaf Agent and Bridge Agent before reaching the Root Agent. This process establishes a complete subscription path. During the message publishing stage, the Publisher sends a Publish message to the Root Agent. The system then applies the LCN hierarchical collaborative network routing algorithm for intelligent route selection through Route_Select. After the route is selected, the message is forwarded through the Bridge Agent to the Leaf Agent. The system finally delivers the message to the Subscriber. This process demonstrates an intelligent routing strategy based on network status and load conditions.

The final part of the process demonstrates the system’s fault tolerance capability. Through continuous health monitoring with HEALTH_CHECK, when a status change is detected, dynamic adjustments to the topology are made via STATUS_UPDATE and TOPOLOGY_UPDATE messages, ensuring the system can adaptively maintain stability and reliability of communication in the event of node failures or network changes.

### 3.2. QoS Routing Algorithm Based on Intelligent Decision Tree

The IQR algorithm integrates adaptive decision trees and a dynamic quality-of-service regulation mechanism. The algorithm extends the static configuration of traditional MQTT routing to a dynamic adjustment model. IQR is not a weighted round-robin (WRR) or feedback control type; rather, it uses reinforcement learning and tree search. The algorithm considers routing selection problems in MQTT communication as Markov Decision Processes. Then it will update the decision strategy by iterative learning.

To explain the decision-making workflow more clearly, the routing process of the IQR algorithm can be summarized in the following steps:

1. The system first collects the current network state *S*. The collected information includes link delay, packet loss rate, broker workload, and message priority;
2. The system then constructs candidate actions by combining broker nodes and QoS levels in the action space *A*;
3. The algorithm evaluates each candidate action according to the reward function R(s,a) and the historical performance dataset *D*;
4. The algorithm finally selects the action with the highest expected reward. The system then performs value backpropagation to update the decision tree structure.

The following subsections describe the state space, action space, reward function, and the four-stage iterative optimization process used in the proposed routing strategy.

The routing decisions of the proposed method are based on real-time network state information. First, collect the following index information from the link: delay, packet loss rate, node load, message attributes, etc. The above are the current network conditions. Then, based on the current state, the algorithm will generate some candidate actions. Each candidate action is a pair of a broker node and a QoS level. Each combination is a possible route and service setup. Next, the algorithm evaluates these candidate actions through a decision-tree-based exploration process. The reward function and historical performance feedback support this evaluation process. The algorithm balances selecting high-performing actions with exploring less frequently selected alternatives. This process helps estimate each candidate’s expected performance. Finally, the algorithm selects the broker–QoS combination with the highest expected reward. This strategy helps balance transmission delay, throughput, and resource consumption.

In the formal definition of the IQR algorithm, the routing decision problem is represented as a quadruple (S,A,T,R). Here, *S* denotes the state space, which includes network environment information and message-related features; *A* denotes the action space, representing the selectable combinations of agent nodes and QoS levels; T:S×A→Π(S) represents the state transition function, describing the probability distribution of the next system state after a specific action is executed; and R:S×A→R represents the reward function used to evaluate decision quality. The state space *S* consists of three parts: network topology status, link performance indicators, and message attributes, which together describe the system’s operating condition. The action space *A* is defined as the Cartesian product of the agent node set and the QoS level set, forming a two-dimensional space for routing decisions.

IQR employs a multi-objective optimization reward function to achieve a coordinated balance among latency, throughput, and resource consumption. Its expression is as follows:

(1) $$R\left(s,a\right)=\alpha \cdot \frac{K}{d}+\beta \cdot p-\gamma \cdot cq$$

In this formulation, *d* represents the message transmission delay in milliseconds. The variable *p* denotes the system throughput in messages per second. The variable cq denotes the additional resource overhead associated with QoS level *q*. The parameter *K* is used as the delay normalization factor. The parameters α, β, and γ represent the weight coefficients for different performance metrics. The reward function uses the reciprocal delay term to encourage lower transmission delay. The function also balances throughput improvement and the reduction of QoS-related resource consumption.

From a practical perspective, the reward function evaluates routing decisions from multiple aspects at the same time. The delay-related term encourages low-latency message transmission. The throughput-related term improves data forwarding efficiency. The cost-related term reduces excessive resource overhead caused by high QoS levels.

Dynamically adjust the weight coefficients according to the various states of traffic, and do not use a fixed set. Different coefficient settings can give different weights to optimisation objectives in practice. Some of the above settings are for low-latency transmission; others aim to enhance communication reliability. The system also uses a small, flexible adjustment module. Periodically update the coefficients in response to changes in the network. For example, when the system detects a large delay or network congestion, it increases the weight of the delay term in the algorithm. The network is relatively stable; therefore, maximise the throughput. Thus, the IQR algorithm can adapt to changes in the network environment while maintaining a relatively low computational cost.

At the implementation level, the IQR algorithm uses a four-stage iterative optimization process based on adaptive decision trees. The process includes node selection, tree expansion, state simulation, and value backpropagation. During the node selection stage, the algorithm introduces a service quality-aware selection function (SQF), which is defined as follows:

(2) $$SQF\left(v\right)=\mu v+E\cdot \sqrt{\frac{\ln(N)}{n_{v}}}+\lambda_{q}\cdot \pi_{t}$$

This formula extends the traditional confidence bound formulation by introducing the QoS adjustment factor $\lambda_{q}$ and the topic priority coefficient $\pi_{t}$. These two factors allow the algorithm to make different decisions for different QoS levels and message categories. In the formula, $\mu_{v}$ represents the average historical reward of node *v*. The variable *N* denotes the number of visits to the parent node. The variable $n_{v}$ indicates the current visit count of node *v*. The parameter *E* represents the exploration parameter. Through this extension, the algorithm can adjust its search strategy according to message priority and QoS requirements. This design helps the algorithm balance exploration and exploitation. The method also improves the adaptability and effectiveness of the decision-making process.

In practice, the SQF function will choose the next candidate node based on both past performance and unexplored routing paths. The algorithm will consider QoS and topic priority when selecting. The above design can select a suitable network path based on the message’s characteristics. The exploitation term $\mu_{v}$ aims to find broker–QoS combinations that have achieved lower delay and higher throughput in the past. Thus, the algorithm will become more stable and converge more smoothly under stable network conditions. At the same time, the exploration term $E\cdot \sqrt{\ln N/n_{v}}$ periodically samples less-visited nodes. Therefore, the risk of being trapped in a locally optimal routing decision due to temporary network abnormalities is reduced. The QoS adjustment factor $\lambda_{q}$ and the topic priority coefficient $\pi_{t}$ further extend the original formulation. The above two factors support the routing process. The factor $\lambda_{q}$ adjusts the exploration rate according to the reliability requirements of a specific QoS level. A relatively larger $\pi_{t}$ indicates that more critical topics are selected for transmission. Thus, the IQR algorithm can balance the use of historically effective paths with the exploration of other paths. The above method can still perform reasonably well in a non-uniform and unstable network environment. It also does not require frequent manual adjustment.

The IQR algorithm maintains a structured performance history database *D*. The database systematically records the communication performance metrics of different agent–topic–QoS combinations:

(3) $$D\left(b,t,q\right)=\left\{\bar{d},m,r,f,\eta \right\}$$

Among these parameters, *b*, *t*, and *q* represent the broker node, message topic, and QoS level, respectively. The parameter $\bar{d}$ denotes the average transmission delay. The variable *m* represents the message count. The variable *r* denotes the number of successfully received messages, while *f* represents the total number of transmitted messages. The parameter η indicates the success rate, where η=r/f. This multidimensional historical record serves as a reference for the simulation stage. The record enables the algorithm to make routing decisions based on historical performance trends. This mechanism also reduces the influence of environmental uncertainty on decision quality.

The IQR algorithm uses a hierarchical three-layer decision tree structure. The first layer represents message topics. The second layer corresponds to candidate agent nodes. The third layer represents selectable QoS levels. With this structure, the algorithm can jointly optimize agent selection and QoS configuration within a single decision process. This design supports collaborative routing and service quality management. Unlike traditional methods that treat QoS as a fixed parameter, the IQR algorithm considers QoS as a dynamic decision variable. This approach improves the system’s adaptability to changing network conditions.

Algorithm A1 shows the formal implementation of the IQR routing process described above. The algorithm is based on the Markov decision process framework. The routing decision process is modeled as a quadruple (S,A,T,R). The state space *S* contains network topology information, link performance indicators, and message characteristics. The action space *A* consists of broker–QoS combinations. During initialization, the algorithm establishes the state and action spaces, as well as the historical performance database. These components provide support for later routing decisions. The core procedure uses a four-stage iterative optimization process based on an adaptive decision tree. The process includes node selection, tree expansion, state simulation, and value backpropagation. During the node selection stage, the Service Quality-aware Selection Function (SQF) evaluates candidate actions. The function considers historical rewards, exploration factors, QoS adjustment coefficients, and topic priorities. During the tree expansion stage, the algorithm enlarges the decision space. During the state simulation stage, the algorithm estimates the expected performance of candidate routing actions. Finally, the value backpropagation stage updates the decision tree according to the evaluation results. These stages correspond to the routing evaluation and optimization process described previously. The reward function jointly considers transmission delay, throughput, and resource consumption. The function also encourages low-latency communication through the reciprocal delay term. The decision tree adopts a hierarchical three-layer structure. The first layer represents message topics. The second layer corresponds to candidate broker nodes. The third layer represents QoS levels. This structure enables the algorithm to optimize routing paths and QoS configurations simultaneously. In addition, the algorithm maintains a structured historical performance database. The database records communication metrics for each broker, topic, and QoS combination. These records provide reference information for state simulation and routing evaluation. Through this iterative learning and optimization process, the IQR algorithm can make adaptive and context-aware routing decisions under dynamic network conditions.

### 3.3. Network Routing Algorithm Based on Hierarchical Tree Structure

The IQR algorithm mainly focuses on the dynamic optimization of broker selection and QoS levels. This design improves message transmission efficiency at the micro level. However, intelligent routing decisions alone cannot fully solve the problems caused by large-scale node deployment and frequent topology changes in complex network environments. Therefore, this section further introduces the Layered Collaborative Network Routing Algorithm (LCN). The LCN algorithm improves system scalability and stability at the macro level through a hierarchical architecture and a decentralized state synchronization mechanism.

The core idea of the LCN algorithm is to organize the MQTT broker network into a multi-level tree structure. The algorithm achieves decentralized state synchronization and topology awareness via a state-propagation protocol.

To explain the routing workflow of LCN more clearly, the overall process can be summarized as follows:

1. The system groups broker nodes into different hierarchical levels according to network proximity measured by RTT;
2. The system periodically propagates node state information through probabilistic neighbor selection;
3. The system maintains local network views and gradually updates global network awareness;
4. The algorithm determines the target hierarchy according to aggregated load information and node health status;
5. The algorithm selects the most suitable node within the chosen hierarchy for message forwarding.

The following subsections describe the formal definitions and detailed mechanisms related to each stage of this process.

This hierarchical organization reduces the complexity of global routing decisions by dividing the routing process into multiple localized optimization tasks. This design follows the divide-and-conquer principle. The approach improves system scalability while maintaining routing efficiency in large-scale distributed environments. Under this framework, system state propagation no longer relies on a centralized node. Instead, nodes gradually spread state information through random communication until the system reaches consistency. Each broker node maintains a local network view. Each node also gradually obtains information about the global topology and resource distribution through periodic information exchange. This mechanism reduces communication overhead and improves system scalability and fault tolerance.

The LCN mechanism organizes distributed broker nodes into a hierarchical structure according to network proximity. Nodes with similar communication latency are grouped into the same level, forming a multi-layer routing architecture. During message forwarding, the system first selects a suitable hierarchy based on overall network conditions, such as load distribution and node health. After the hierarchy is selected, the system chooses the target node within that level. The selection process considers factors such as node availability, load condition, and connectivity. This hierarchical routing strategy reduces routing complexity. The strategy also improves system scalability and fault tolerance.

From a theoretical perspective, the hierarchical structure reduces the complexity of global state management. In a fully decentralized flat architecture, each node must maintain the state information of all other nodes. This design incurs $O(N^{2})$ communication overhead. The LCN algorithm organizes nodes into a multi-level tree according to RTT proximity. With this structure, frequent state synchronization mainly occurs within the same hierarchy. The system uses lightweight summary information for communication between different levels. This design reduces the overall communication complexity to approximately O(NlogN). In addition, the distance-preference-based propagation mechanism allows physically closer nodes to exchange state information more frequently. These nearby nodes are more likely to experience similar network conditions. Therefore, the shared information becomes more relevant, and the system reduces unnecessary long-distance transmissions.

In terms of theoretical modeling, LCN is formalized as a five-tuple (N,L,S,C,R), where $N=\{n_{1},n_{2},\ldots ,n_{n}\}$ represents the set of *n* agent nodes in the system; L:N→{1,2,…,k} is a mapping function from nodes to *k* levels; $S(n_{i},t)$ represents the state information of node $n_{i}$ at time *t*, including load, health status, topic distribution, etc.; *C* defines the communication rules between nodes; and *R* describes the message routing strategy based on state and level information.

In the state propagation model *C*, node $n_{i}$ randomly selects *m* target nodes for state exchange within each time interval Δt, where *m* is the diffusion factor. The probability distribution formula for the selection of target nodes is as follows:

(4) $$Q\left(n_{j}|n_{i}\right)=\rho \cdot \exp\left(-\frac{v(n_{i},n_{j})}{\sigma }\right)$$

Here, $v(n_{i},n_{j})$ represents the network distance (such as RTT) between nodes $n_{i}$ and $n_{j}$, σ is the network sensitivity factor, and ρ is the normalization parameter to ensure that $\sum_{j}Q\left(n_{j}|n_{i}\right)=1$. This distance-preference-based random selection mechanism increases the probability of communication between nearby nodes. As a result, the system can reduce the overhead of long-distance communication. At the same time, the mechanism maintains stable random propagation behavior and system robustness.

When node $n_{i}$ sends status information to node $n_{j}$, node $n_{j}$ integrates the received state $S(n_{i},t)$ into its local state information. To support this process, the LCN algorithm uses a status merging method based on version information, which is defined as follows:

(5) $$S\left(n_{j},t+1\right)=F\left(S\left(n_{j},t\right),S\left(n_{i},t\right)\right)$$

Here, the merge function *F* performs different operations for different types of state information. The system updates the topic distribution data through the maximum value rule. The system combines health-related indicators using a weighted average. The system updates the version number by preserving the larger value. This design increases the efficiency of information propagation and lowers the possibility of conflicts and inconsistencies during synchronization.

Another important module of the LCN algorithm is the hierarchical routing strategy *R*. The system assigns node $n_{i}$ to different hierarchical levels $L(n_{i})$ according to the network RTT. The level division function is defined as follows:

(6) $$L\left(n_{i}\right)=\left\lfloor k\cdot \frac{\mathrm{RTT}\left(n_{i}\right)-{\mathrm{RTT}}_{\min}}{{\mathrm{RTT}}_{\max}-{\mathrm{RTT}}_{\min}+\varepsilon }\right\rfloor +1$$

Among these parameters, $\mathrm{RTT}(n_{i})$ represents the round-trip delay of node $n_{i}$. The parameters ${\mathrm{RTT}}_{\min}$ and ${\mathrm{RTT}}_{\max}$ denote the minimum and maximum RTT values in the network, respectively. The parameter ε is a small positive constant that prevents division-by-zero problems. The symbol ⌊·⌋ denotes the floor operation. With this mechanism, the system groups nodes with similar network characteristics into the same hierarchy. This process forms a multi-level logical tree structure.

To improve stability under dynamic network conditions, the LCN mechanism introduces a dual-layer stabilization strategy. The strategy combines time series smoothing with a hysteresis-based update policy.

For time series smoothing, the RTT value used in Equation (6) is not an instantaneous measurement but an exponentially weighted moving average (EWMA):

(7) $${\mathrm{RTT}}_{\mathrm{smooth}}\left(n_{i},\tau \right)=\varphi \cdot {\mathrm{RTT}}_{\mathrm{instant}}\left(n_{i},\tau \right)+\left(1-\varphi \right)\cdot {\mathrm{RTT}}_{\mathrm{smooth}}\left(n_{i},\tau -1\right)$$

where φ=0.2 is the smoothing factor. This low-pass filter suppresses high-frequency noise caused by transient network bursts, ensuring that only sustained changes affect hierarchy assignment.

To improve the stability of hierarchy switching, the system further introduces a hysteresis-based update mechanism. The system does not adjust the hierarchy according to instantaneous RTT fluctuations. Instead, the system evaluates the smoothed RTT over several consecutive periods before triggering a hierarchy change. A node is promoted to a lower-latency level only when ${\mathrm{RTT}}_{\mathrm{smooth}}\left(n_{i}\right)<T_{\mathrm{promote}}\left(L\right)-\Delta_{h}$. A node is demoted to a higher-latency level only when ${\mathrm{RTT}}_{\mathrm{smooth}}\left(n_{i}\right)>T_{\mathrm{demote}}\left(L\right)+\Delta_{h}$. In this formulation, $\Delta_{h}$ represents the guard interval. In addition, the system performs hierarchy reassignment only when the corresponding condition remains valid for at least *K* consecutive evaluation periods. This mechanism reduces short-term oscillations and avoids unnecessary updates to global routing tables under dynamic network conditions. At the same time, the system can still respond to persistent long-term performance changes.

During message routing, the LCN algorithm first determines the target hierarchy. The algorithm then selects a specific broker node within that level. The selection probability of each hierarchy depends on node health conditions and load distribution. The calculation is defined as follows:

(8) $$P\left(i\right)=\alpha_{i}\cdot \left(H_{i}^{k}\right)\cdot \left(1-L_{i}^{j}\right)$$

The routing process uses a two-stage decision strategy. First, the algorithm selects the target hierarchy based on aggregated information, such as the average node health status and load conditions at each level. After the hierarchy is selected, the algorithm applies a weighted evaluation function to choose a specific node within that level. This hierarchical routing approach improves routing efficiency and reduces the overhead caused by global optimization.

Here, *i* represents the hierarchical index, $\alpha_{i}$ is the hierarchical weight coefficient, $H_{i}$ is the average health of level *i*, $L_{i}$ is the average load, and *k* and *j* are weight parameters that control the influence of the two on the selection probability. Within the hierarchy, node selection is based on the following comprehensive score:

(9) $$V\left(n_{i}\right)=\omega_{1}\cdot h\left(n_{i}\right)+\omega_{2}\cdot \left(1-l\left(n_{i}\right)\right)+\omega_{3}\cdot c\left(n_{i}\right)$$

Among them, $h(n_{i})$ represents the node health, $l(n_{i})$ represents the load level, and $c(n_{i})$ represents the connectivity (the number of connections with other nodes). $\omega_{1}$, $\omega_{2}$, and $\omega_{3}$ are weight coefficients, satisfying $\omega_{1}+\omega_{2}+\omega_{3}=1$. The probability of a node being selected is proportional to its score.

In addition, LCN introduces a topic-based dynamic routing table mechanism. The system maintains a mapping M:T→P(N), associating each topic in *T* with a set of broker nodes. The routing table is updated dynamically according to the subscription information obtained through the state propagation protocol, and the update rules are defined as follows:

(10) $$M\left(t,\tau +1\right)=\left\{n_{i}\in N\;|\;B\left(n_{i},t,\tau \right)>0\;\vee \;\left(n_{i}\in M\left(t,\tau \right)\;\wedge \;\neg U\left(n_{i},\tau \right)\right)\right\}$$

Here, $B(n_{i},t,\tau )$ represents the number of subscribers for topic *t* at node $n_{i}$ at time τ. The function $U(n_{i},\tau )$ is a Boolean function that indicates whether node $n_{i}$ is in an unhealthy state at that time. With this mechanism, the system forwards messages only to broker nodes that maintain subscriptions for the corresponding topic. This design reduces unnecessary network traffic.

The system also includes a health monitoring and fault detection module. Through the health assessment function $H:N\times R^{+}\to \left[0,1\right]$, the operational status of a node at a certain moment is mapped to a score within the [0,1] interval:

(11) $$H\left(n_{i},\tau \right)=\delta_{1}\cdot \exp\left(-\mu \cdot (\tau -T\left(n_{i}\right))\right)+\delta_{2}\cdot R\left(n_{i}\right)+\delta_{3}\cdot A\left(n_{i}\right)$$

Here, $T(n_{i})$ represents the most recent state update time of node $n_{i}$. The parameter μ denotes the time decay coefficient. The parameter $R(n_{i})$ represents the response rate, while $A(n_{i})$ indicates node availability. The parameters $\delta_{1}$, $\delta_{2}$, and $\delta_{3}$ are weighting factors that satisfy $\delta_{1}+\delta_{2}+\delta_{3}=1$. When the health score of a node falls below the threshold $\theta_{h}$, the system activates the fault handling mechanism. The mechanism removes the node from the routing table and reallocates its topic-related tasks.

By combining state propagation with hierarchical routing, the LCN algorithm provides several important features. The mechanism supports sublinear growth in communication overhead as the number of nodes increases. The mechanism also adapts to topology changes and remains resilient to node failures. In addition, the system supports decentralized state maintenance and efficient hierarchy-based scheduling. This mechanism is suitable for large-scale IoT deployments with geographically distributed nodes and complex network conditions. A typical case is a smart city and an Industrial Internet of Things (IIoT) platform. The LCN algorithm is capable of processing the same data differently according to the demand for low latency. The system will send out urgent control messages at a lower delay. At the same time, the system will be able to handle large amounts of general data more easily. The above characteristics can provide technical support for large-scale and high-performance distributed MQTT systems.

Algorithm A2 describes the detailed implementation of the LCN hierarchical collaborative routing mechanism. The algorithm first initializes node states and constructs the network distance matrix through RTT measurements. To improve stability under dynamic network conditions, the system smooths RTT values by using an exponentially weighted moving average (EWMA). The system also controls hierarchy switching through a hysteresis-based update mechanism. According to the smoothed RTT results, the system divides nodes into different levels to form the hierarchical routing structure.

During operation, nodes periodically exchange information through the state propagation mechanism. To reduce control-plane overhead in large-scale network deployments, the system uses probabilistic, localized synchronization rather than full-table synchronization. In practice, each node exchanges state information only with a limited number of neighboring nodes selected according to a probability distribution. The system does not broadcast complete subscription mappings across the entire network.

In addition, the propagated information mainly contains incremental state updates instead of complete routing tables. Each node maintains lightweight version information for its local subscription state. The node transmits only the entries updated after the previous synchronization with a neighboring node. With this design, repeated local exchanges propagate only the modified parts of the subscription mapping. As a result, the system can gradually obtain global state awareness while avoiding the overhead introduced by full-table synchronization.

During steady-state operation, subscription patterns usually remain relatively stable. Under this condition, the control traffic of each node mainly depends on the number of updated topics Δ|T|. In most cases, this value is much smaller than the total number of active topics |T|. As a result, the LCN mechanism avoids the scalability problems found in centralized or full-synchronization methods. The mechanism can also maintain efficient operation in large-scale and high-concurrency network environments.

Each node first calculates a probability distribution for neighbor selection. Then the node selects a subset of its neighbours to exchange state information and thus achieves decentralised network awareness. At the same time, a health assessment function is running continuously in the node. The function considers factors such as response time, reliability, and availability. According to the above factors, the system will calculate a health score and identify unhealthy nodes.

Select a destination tier based on the combined health and capacity of all tiers during message routing. A hierarchy has been formed, and now all nodes in that level will be assigned a total score by the algorithm. The components of the score are node health, load level and connection. Then, the system will forward the message to the node with the highest score. The system will also update the topic routing table dynamically. The table will ensure that the messages are delivered only to nodes that have subscribed to the corresponding topics. This Design will reduce the network load.

Hierarchical collaboration can distribute the load of the LCN mechanism more evenly. A mechanism for improved resource utilisation through decentralized state synchronisation and adaptive fault handling. The System can still work normally in a fluctuating network environment. This Design can be applied to a large-scale distributed environment that has continually changing topology and network conditions.

## 4. Experimental Results and Discussion

We will conduct a series of experiments to test the new DAIS-MQTT method in this section. The two sections of the experiment are: First, we will test how adaptable and stable DAIS-MQTT is in three network conditions: small, medium and large. Second, compare DAIS-MQTT with other distributed MQTT solutions. Compare the end-to-end delay, system throughput and message delivery reliability of these.

To ensure the validity of the experiment, a full-featured experimental platform that mimics the communication environment of a distributed Internet of Things (IoT) will be established in this paper. It is a high-performance platform that has been equipped with an Intel Core i9-14900K (5.8 GHz) CPU, 64 GB of DDR5 RAM and a 2 TB NVMe SSD. There are 9 MQTT broker nodes in the distributed communication system written in Python 3.13. These nodes cooperate to receive, process and forward messages in a hierarchical logical structure. Publisher and subscriber clients are also implemented as Python scripts. The generated message streams of these clients have different priorities, sizes and types according to preset strategies. Thus, this system can be tested under multiple sets of stress. To ensure reproducibility and clarity of the experimental evaluation, the key configuration parameters are summarized in Table 1.

Based on the above experimental configuration, to accurately reproduce the complexity of heterogeneous network environments, this study introduces the Clumsy Network Interference tool. By controllably adjusting the link delay and packet loss rate, three types of network environments are constructed to cover typical industrial Internet of Things scenarios. The experimental evaluation system adopts multi-dimensional indicators, including average message transmission delay, system throughput (messages/second), message transmission success rate, and QoS level distribution.

In addition, to objectively compare the performance of DAIS-MQTT, this study selects three representative MQTT implementations as benchmark schemes: the traditional single-agent model (Mosquitto), MQTT-ST based on static topology [40], and TOD-MQTT, which considers network topology optimization [10]. All systems were tested under the same hardware and network conditions to ensure the fairness and reliability of the comparison results.

### 4.1. Adaptive Analysis

This section systematically evaluates the performance of DAIS-MQTT in three different network environments, with a focus on the adaptive capabilities of the IQR intelligent routing algorithm and the LCN hierarchical collaboration network mechanism under varying network conditions. The experiments precisely control network parameters (latency and packet loss rate) to construct different network environments: small-scale network (10–50 ms latency, 0–1% packet loss rate), medium-scale network (50–200 ms latency, 1–5% packet loss rate), and large-scale network (200–500 ms latency, 5–15% packet loss rate).

**Figure 3:**
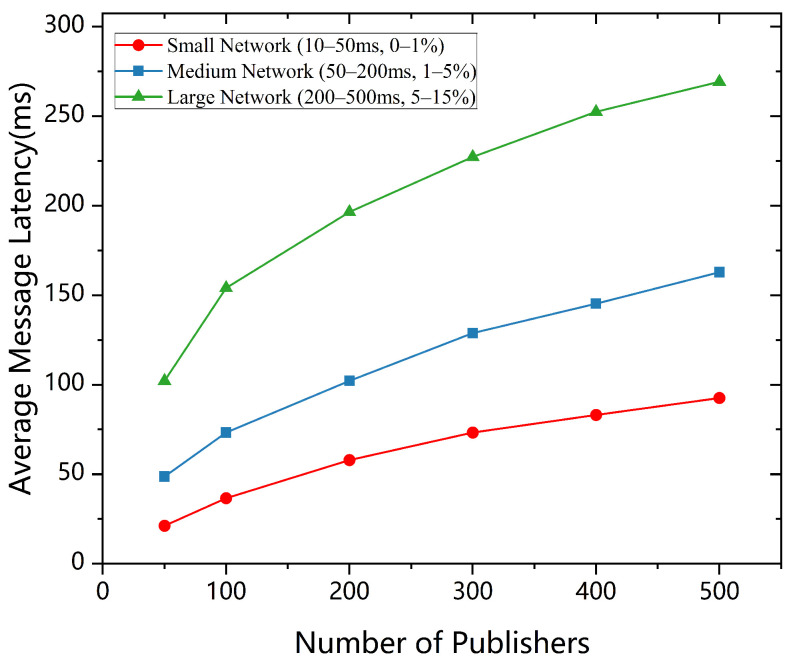
The average message delay of DAIS-MQTT in different network environments.

Figure 3 presents the variation in the average message transmission delay of DAIS-MQTT under three network environments. As network conditions become more complex, the system still controls the increase in delay through adaptive routing and dynamic QoS adjustment. In the small-scale network scenario, the delay increases from 21 ms with 50 publishers to 91 ms with 500 publishers. This result shows relatively stable growth as the traffic load increases. The IQR mechanism primarily drives this behavior. The mechanism dynamically adjusts routing paths and QoS configurations to reduce congestion and retransmissions. In medium-scale and large-scale network environments, the delay remains within an acceptable range even under higher latency and packet-loss conditions. This result indicates that the LCN hierarchical mechanism reduces routing overhead and improves routing efficiency during message forwarding.

**Figure 4:**
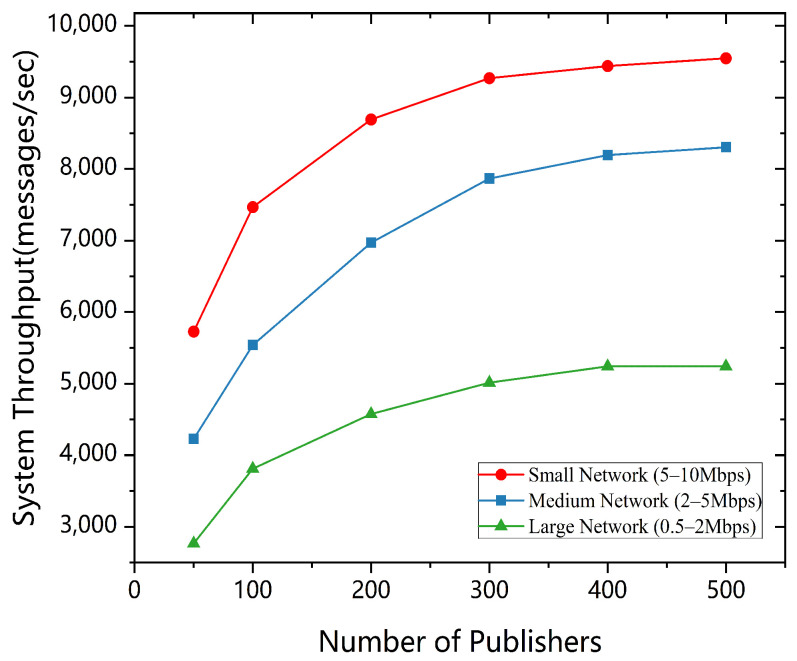
The system throughput of DAIS-MQTT in different network environments.

Figure 4 shows the throughput performance of DAIS-MQTT under three different network environments. The results indicate that system throughput increases almost linearly with the number of publishers. This behavior demonstrates good system scalability. Even in the large-scale network scenario, the throughput reaches 5250 messages per second. This value is approximately 55% of the peak throughput achieved in the small-scale environment. The performance improvement mainly comes from the combined operation of IQR and LCN. The IQR mechanism improves resource utilization by dynamically adjusting QoS. At the same time, the LCN mechanism reduces communication overhead through hierarchical routing. These mechanisms allow the system to maintain efficient message transmission under constrained network conditions.

**Figure 5:**
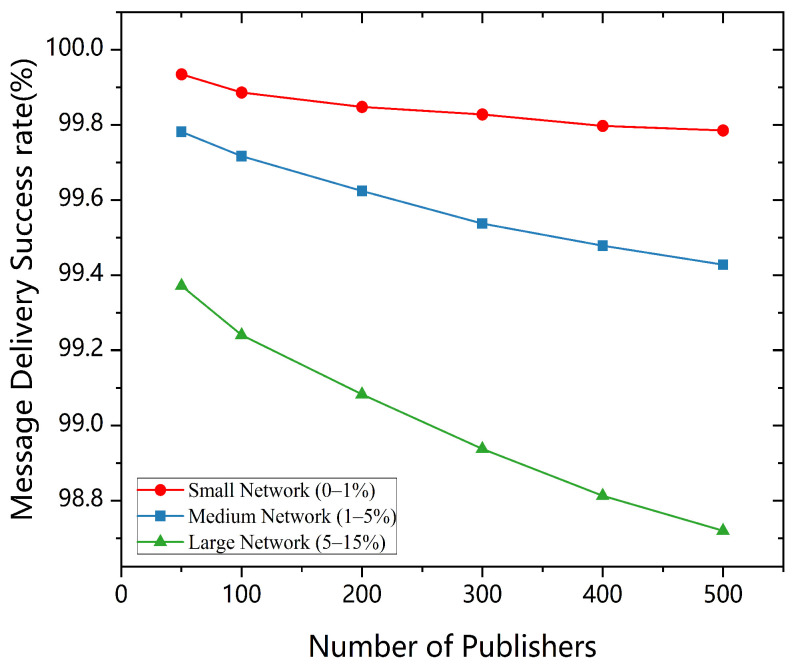
The message transmission reliability of DAIS-MQTT in different network environments.

Figure 5 presents the message delivery reliability of DAIS-MQTT under different network conditions. The system maintains a relatively high delivery success rate in all scenarios. Even in large-scale networks under heavy load, the delivery reliability reaches 98.72%. This value remains significantly higher than the baseline network reliability. This improvement mainly comes from adaptive QoS adjustment and routing optimization. These mechanisms improve communication reliability under unstable network conditions. Although the delivery rate decreases slightly as the number of publishers increases, the overall reduction remains limited. This result indicates that the system remains robust under network fluctuations.

**Figure 6:**
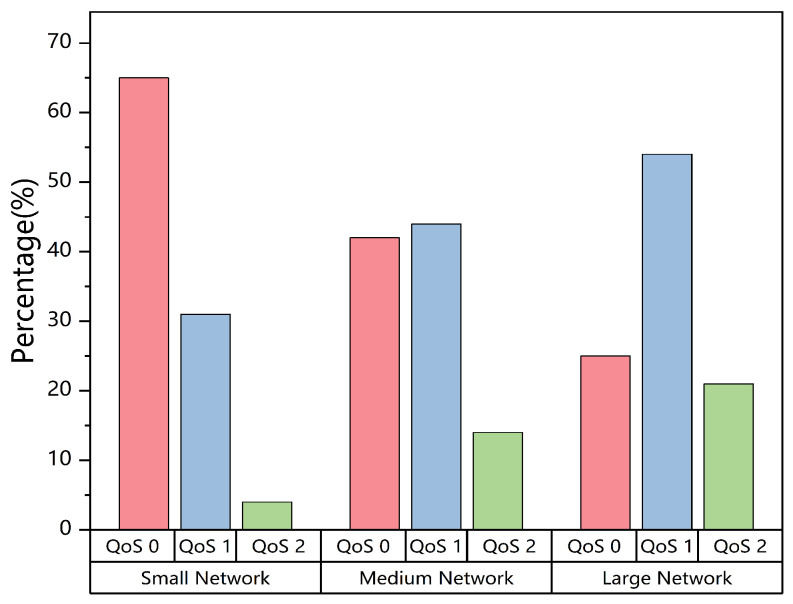
The distribution of QoS levels of DAIS-MQTT in different network environments.

Figure 6 shows the distribution of QoS levels selected by DAIS-MQTT under different network conditions. In small-scale network environments, QoS 0 (65%) and QoS 1 (31%) account for the majority of message transmissions. This distribution helps reduce communication overhead and maintain high throughput. As network conditions become more challenging, the system gradually increases the use of higher-reliability QoS levels. In large-scale networks, the proportion of QoS 1 increases to 54%, while QoS 2 reaches 21%. This result demonstrates the IQR mechanism’s adaptive capability. The mechanism dynamically balances transmission reliability and resource consumption according to current network conditions.

Overall, the experimental results show that DAIS-MQTT maintains stable performance under different network environments. The IQR mechanism supports adaptive routing and QoS adjustment according to real-time network conditions. The LCN mechanism improves system scalability through hierarchical scheduling and decentralized coordination. By combining these two mechanisms, the system maintains a balance among transmission delay, throughput, and communication reliability under varying network conditions.

### 4.2. Performance Comparison Analysis

After evaluating the adaptability and stability of DAIS-MQTT under different network environments, this section further conducts comparative experiments with three representative MQTT implementations: MQTT, MQTT-ST, and TOD-MQTT. Conduct experiments to verify the advantages of the proposed method in terms of transmission delay, throughput and message reliability. The comparison experiments will be conducted in a medium-scale network environment. All the above methods use the same load pattern and evaluation indicators. Therefore, this design can ensure the fairness and comparability of the experimental results.

Increase the load in the experiment gradually using the load-test method. The number of publishers has risen from 10 to 500 gradually. Therefore, the changes in performance for all the systems under different loads can be investigated. All tested systems ran on the same hardware platform and network environment. To reduce the influence of random fluctuations, each experiment was repeated five times. The evaluation used the average result of these runs. The main performance metrics included average message transmission delay, system throughput, and message delivery reliability.

**Figure 7:**
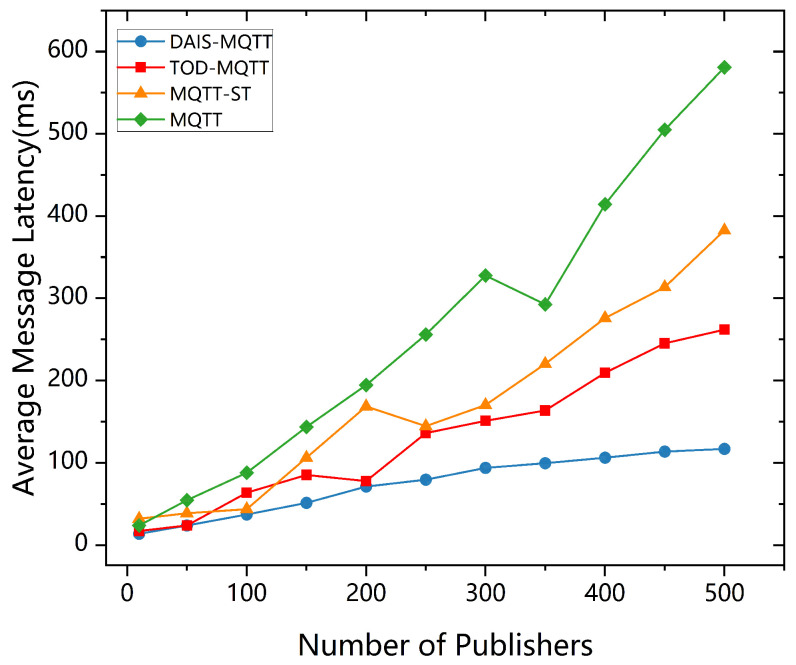
Comparison of average message transmission delay across different systems with increasing numbers of publishers.

Figure 7 compares the average message transmission delay of different systems as the number of publishers increases. Under low-load conditions with fewer than 50 publishers, all systems show similar delay performance. As the load increases, the performance differences among the systems become more noticeable. When the number of publishers reaches 200, the MQTT delay increases to 195 ms. MQTT-ST and TOD-MQTT achieve delays of 168 ms and 78 ms, respectively. In comparison, DAIS-MQTT maintains a 71 ms delay. This value is about 9% lower than TOD-MQTT and 64% lower than MQTT. Under high-load conditions with 500 publishers, the DAIS-MQTT delay remains below 118 ms. In contrast, the delays of the other systems exceed 250 ms. The rapid growth in MQTT is mainly due to the centralized broker bottleneck. As the overall load increases, queuing delays become significantly higher. MQTT-ST depends on a relatively static topology and therefore cannot adapt effectively to changes in congestion. TOD-MQTT improves topology management, but the method does not support dynamic QoS adjustment. As a result, messages compete for resources in the same way under heavy traffic conditions. By comparison, DAIS-MQTT combines the joint routing and QoS-optimization capabilities of IQR with the hierarchical scheduling mechanism of LCN. The system considers both load conditions and node health status during routing. This design helps reduce delay growth under high-load conditions.

**Figure 8:**
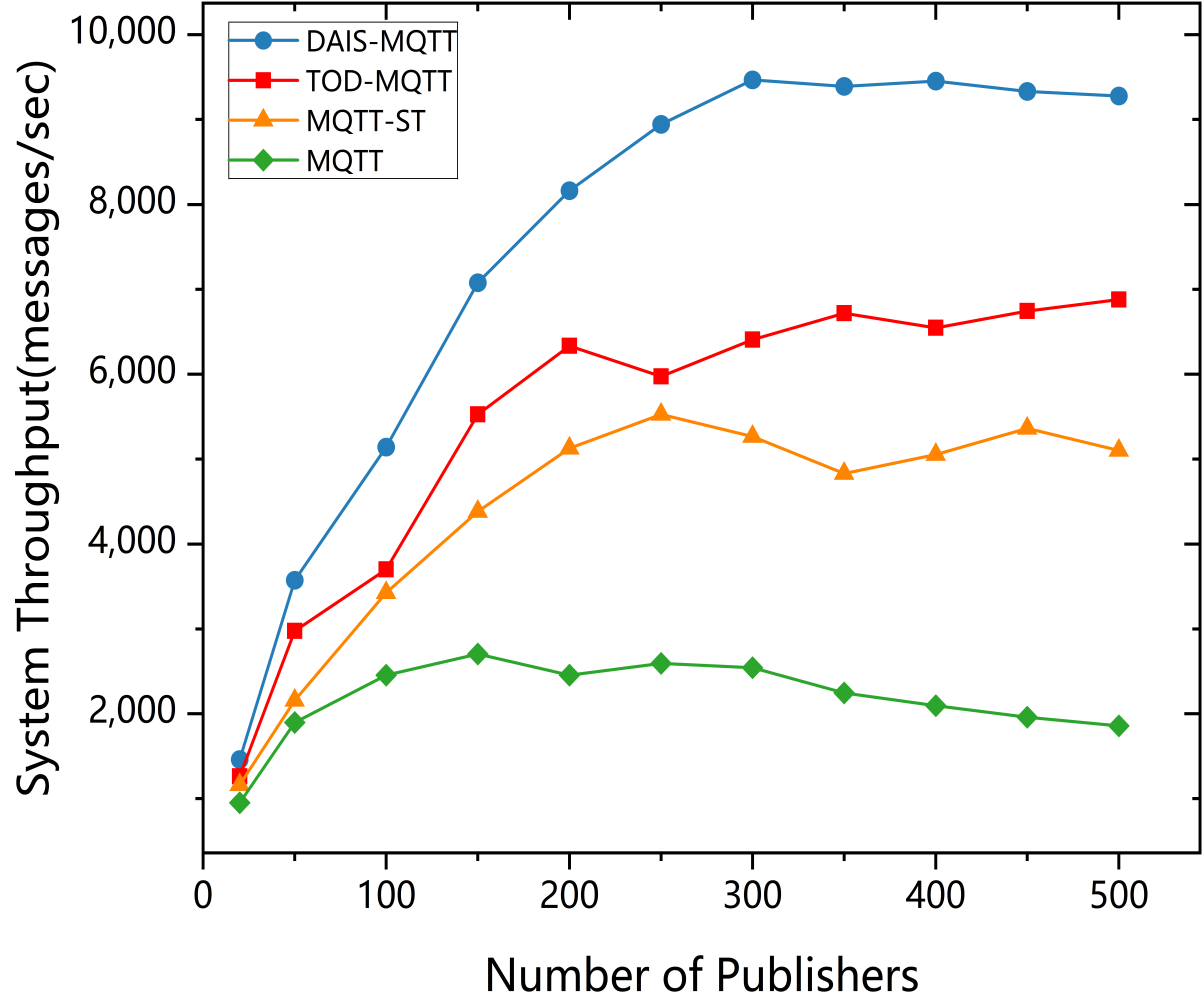
Comparison of throughput across different systems with increasing numbers of publishers.

Figure 8 presents the throughput variation of different systems as the number of publishers increases. The results show that DAIS-MQTT maintains higher throughput under different load conditions. When the number of publishers reaches 100, DAIS-MQTT achieves a throughput of 5150 messages per second. This value exceeds MQTT (2450 messages per second), MQTT-ST (3450 messages per second), and TOD-MQTT (3680 messages per second). As the load further increases to 300 publishers, the throughput of DAIS-MQTT reaches a peak value of 9480 messages per second. The main reasons for the throughput limitations of the baseline methods are their architectural characteristics. MQTT uses a single central broker, so it is more likely to be congested. MQTT-ST and TOD-MQTT are multi-broker systems that cannot automatically distribute the load when some brokers are overloaded. DAIS-MQTT’s LCN mechanism continuously distributes messages to nodes with lower load. At the same time, the IQR mechanism selects the best route based on the network status. The combination will improve the use of all resources and increase the system’s total throughput. The minor drop in throughput after 300 publishers is mainly due to the increased coordination overhead of the LCN state.

**Figure 9:**
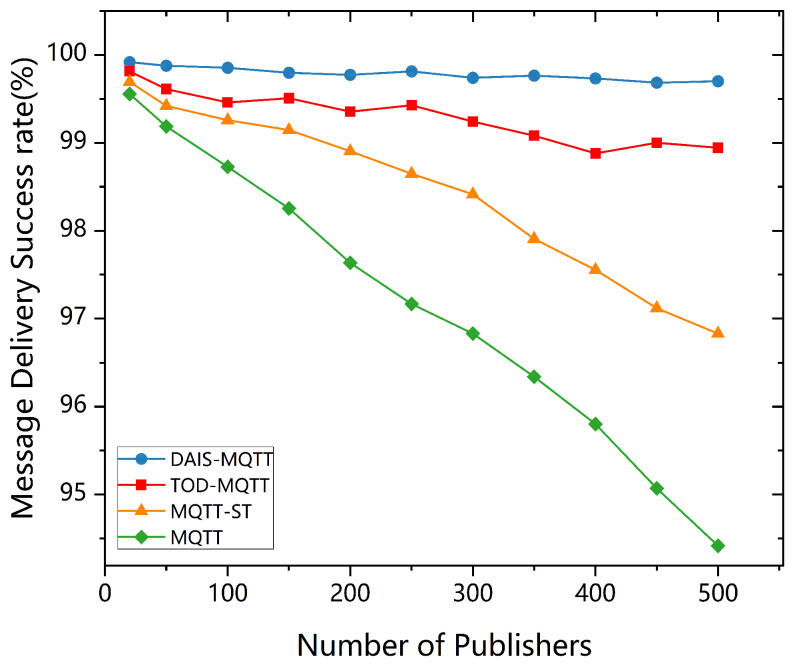
Comparison of message transmission reliability across different systems under varying load conditions.

Figure 9 shows the message-delivery reliability of each system under different load conditions. DAIS-MQTT has achieved a delivery success rate of 99.79% in the medium-scale network scenario with 200 publishers. The above two are 97.65% and 98.95%, respectively. The result is slightly higher than TOD-MQTT (99.38%). With more than 300 publishers, all systems are now experiencing performance issues. DAIS-MQTT has the least drop and is thus more stable. Standard MQTT is more prone to single-point failures, leading to message loss under high load. MQTT-ST uses a reactive fault recovery strategy, which causes temporary reliability reductions during switchover. TOD-MQTT cannot improve delivery guarantees when routing paths become degraded. DAIS-MQTT combines the proactive health monitoring mechanism of LCN with the adaptive QoS adjustment capability of IQR. With this design, the system maintains high communication reliability. The delivery rate remains above 99.5%, while the other methods’ rates fall below 98.5%.

The comparison results show that DAIS-MQTT performs better than the other MQTT-based methods in terms of transmission delay, throughput, and message reliability. The proposed method reduces communication delay through adaptive routing and hierarchical scheduling. The system also maintains higher throughput under different load conditions. At the same time, the distributed architecture improves system scalability. In addition, the combination of adaptive QoS adjustment and routing optimization helps the system maintain high reliability even when network load changes significantly.

Although the above results verify the effectiveness of DAIS-MQTT, the current experimental evaluation still has several limitations. The experiments were conducted in a simulated environment. The system used only 9 broker nodes deployed on a single high-performance machine. In addition, the Clumsy network interference tool can emulate latency and packet loss, but the adopted network settings remain relatively stable. The actual industrial network environment is more complex than the above. Burst interference, node mobility and heterogeneous link conditions can all occur at the same time in practice. Therefore, the experiments have clearly shown the superior performance of DAIS-MQTT. However, the absolute performance results will be different in large-scale deployments of physically distributed devices and more complex network environments.

Despite the above deficiencies, the experiment’s environment is still controlled and repeatable. The above method can compare all other ways reasonably and consistently. Therefore, the observed performance trends remain meaningful for evaluating the effectiveness of the proposed IQR and LCN mechanisms. The results are especially useful for analyzing adaptive routing behavior and QoS-aware optimization under increasing network load.

## 5. Conclusions

With the rapid expansion of IoT applications, traditional MQTT systems face limitations in scalability and fault tolerance due to centralized brokers, as well as static QoS configurations that struggle to adapt to dynamic environments, making it difficult to meet the high concurrency demands of large-scale distributed environments. This paper proposes DAIS-MQTT, which achieves network topology awareness and distributed message processing through the collaborative work of the network infrastructure layer, broker collaboration layer, and intelligent decision-making layer. By using the intelligent decision tree-based QoS routing algorithm (IQR) for adaptive learning and optimization of broker selection and QoS configuration, it enhances the adaptability of communication paths and service quality. On this basis, the hierarchical tree structure-based network routing algorithm (LCN) realizes efficient and fault-tolerant multi-node collaborative communication through decentralized state propagation and hierarchical scheduling. DAIS-MQTT integrates intelligent decision-making and distributed communication principles in its design, significantly improving system throughput and message transmission delay performance, and demonstrating excellent scalability and environmental adaptability.

Although the experimental results demonstrate the effectiveness of DAIS-MQTT across different network conditions, the current evaluation is still conducted in a controlled simulation environment. In the experimental setup, the distributed broker system consisting of nine broker nodes was deployed on a single high-performance machine to ensure reproducibility and consistent performance evaluation. Therefore, the current results primarily demonstrate the performance trends, adaptability, and coordination capabilities of the proposed framework rather than the full scalability of physically distributed industrial deployments. Further validation in geographically distributed and large-scale industrial environments with heterogeneous edge devices and real network infrastructures will be an important direction for future research.

In the future, new distributed optimization methods will be introduced based on the DAIS-MQTT architecture to further enhance the system’s intelligent decision-making capabilities. It is planned to integrate advanced path optimization technologies to improve the selection efficiency of IQR in complex network environments. At the same time, the integration of multi-node coordination mechanisms into the LCN framework will be explored to enhance the synchronization and consistency among agents. By comparing the performance of different optimization strategies in the DAIS-MQTT system, the system will evaluate their applicability and stability advantages in dynamic industrial environments.

Future research will continue to refine and extend the proposed DAIS-MQTT communication framework toward more adaptive and intelligent distributed coordination. On the one hand, more advanced intelligent scheduling mechanisms can be further explored by integrating dynamic network perception and learning-based decision strategies, so as to improve the global optimization capability of QoS-aware routing and enhance load balancing efficiency under highly dynamic and large-scale industrial scenarios. In particular, the joint optimization of the IQR and LCN mechanisms can be further strengthened to achieve more efficient collaborative routing and resource allocation among distributed brokers.   

## Figures and Tables

**Table 1:** Experimental configuration parameters.

Parameter	Value
Number of broker nodes	9
Number of topics	20
Message size distribution	80% small (128 bytes), 20% large (1024 bytes)
Reward weight coefficients	α=0.5, β=0.3, γ=0.2
Experiment duration per run	600 s
Sampling interval	5 s

## Data Availability

The original contributions presented in this study are included in the article. Further inquiries can be directed to the corresponding author.

## Appendix A

**Algorithm A1** Intelligent QoS Routing Based on Decision Tree (IQR)
**Input:** Message msg, topic set *T*, broker set *B*, QoS levels *Q*
**Output:** Optimal broker–QoS pair $\left(b^{*},q^{*}\right)$
1: Initialize state space *S*
2: Initialize action space A=B×Q
3: Initialize database D(b,t,q)=∅
4: **function** RewardFunction(s,a)
5: Parse a→(b,q)
6: Estimate delay *d*, throughput *p*, cost $c_{q}$
7: **return** $\alpha \cdot \left(K/d\right)+\beta \cdot p-\gamma \cdot c_{q}$
8: **end function**
9: **function** SQF(*v*)
10: $\mu_{v}\leftarrow$ average reward
11: N← parent visits, $n_{v}\leftarrow$ node visits
12: $\lambda_{q}\leftarrow$ QoS factor, $\pi_{t}\leftarrow$ priority
13: **return** $\mu_{v}+E\cdot \sqrt{\ln\left(N\right)/n_{v}}+\lambda_{q}\cdot \pi_{t}$
14: **end function**
15: **function** SimulateState(s,a)
16: Execute *a*, observe $\left(s^{\prime },r\right)$
17: **return** $\left(s^{\prime },r\right)$
18: **end function**
19: **function** DecisionTree(msg,t)
20: Initialize root
21: **for** i=1 to max_iterations **do**
22: v← SelectNode using SQF
23: **if** *v* not fully expanded **then**
24: $v^{\prime }\leftarrow$ ExpandNode(*v*)
25: **else**
26: $v^{\prime }\leftarrow v$
27: **end if**
28: $(s^{\prime },r)\leftarrow$ SimulateState(state($v^{\prime }$),action($v^{\prime }$))
29: UpdateTree($v^{\prime }$, *r*)
30: **end for**
31: $(b^{*},q^{*})\leftarrow$ best child
32: Update $D(b^{*},t,q^{*})$
33: **return** $\left(b^{*},q^{*}\right)$
34: **end function**
35: $(b^{*},q^{*})\leftarrow$ DecisionTree(msg,topic(msg))
36: **return** $\left(b^{*},q^{*}\right)$

**Algorithm A2** Layered Cooperative Network Routing (LCN)
**Input:** Node set *N*, layer count *k*, diffusion factor *m*, topic set *T*
**Output:** Optimized routing *R* and scheduling strategy *S*
1: Initialize node states
2: Measure RTT matrix *D*
3: Compute ${\mathrm{RTT}}_{smooth}\left(n_{i}\right)$ using EWMA (Equation (7))
4: **for** each $n_{i}\in N$ **do**
5: **if** ${\mathrm{RTT}}_{smooth}\left(n_{i}\right)$ persistently violates hysteresis thresholds for *K* intervals **then**
6: $L\left(n_{i}\right)\leftarrow \left\lfloor k\cdot \frac{{\mathrm{RTT}}_{smooth}\left(n_{i}\right)-RTT_{\min}}{RTT_{\max}-RTT_{\min}+\epsilon }\right\rfloor +1$
7: **end if**
8: **end for**
9: **function** PropagateState($n_{i}$)
10: Compute selection probability $Q(n_{j}|n_{i})$ based on network proximity and load
11: Select *m* nodes based on *Q* and propagate incremental state updates
12: **return** updated state
13: **end function**
14: **function** EvaluateHealth($n_{i}$)
15: $H\left(n_{i}\right)\leftarrow \delta_{1}e^{-\mu (\tau -T\left(n_{i}\right))}+\delta_{2}R\left(n_{i}\right)+\delta_{3}A\left(n_{i}\right)$
16: **if** $H\left(n_{i}\right)<\theta_{h}$ **then**
17: Mark $n_{i}$ as unhealthy
18: **end if**
19: **return** $H(n_{i})$
20: **end function**
21: **function** SelectRoutingNode(msg,t)
22: **for** i=1 to *k* **do**
23: $P\left(i\right)\leftarrow \alpha_{i}\cdot H_{i}\cdot \left(1-L_{i}\right)$
24: **end for**
25: Select target level $i^{*}$
26: $N_{L}\leftarrow \left\{n_{i}|L\left(n_{i}\right)=i^{*}\right\}$
27: **for** each $n_{i}\in N_{L}$ **do**
28: $V\left(n_{i}\right)\leftarrow \omega_{1}h\left(n_{i}\right)+\omega_{2}\left(1-l\left(n_{i}\right)\right)+\omega_{3}c\left(n_{i}\right)$
29: **end for**
30: **return** $\arg\max V(n_{i})$
31: **end function**
32: Initialize M(t)=∅, τ=0
33: **while** system is running **do**
34: **for** each $n_{i}\in N$ **do**
35: PropagateState($n_{i}$)
36: EvaluateHealth($n_{i}$)
37: **end for**
38: Update topic routing table M(t)
39: **for** each message (msg,t) **do**
40: $n^{*}\leftarrow$ SelectRoutingNode(msg,t)
41: Route message via $n^{*}$
42: **end for**
43: τ←τ+1
44: **end while**
45: **Return** R,S
